# Spiritual rehabilitation of affected people after natural disasters: a protocol for a systematic review

**DOI:** 10.5249/jivr.v15i2.1830

**Published:** 2023-07

**Authors:** Bayram Nejati-Zarnaqi, Davoud Khorasani-Zavareh, Sanaz Sohrabizadeh, Mohtasham Ghaffari, Siamak Sabour, Reza Mohammadi

**Affiliations:** ^*a*^ Department of Health in Disasters and Emergencies, School of Public Health and Safety, Shahid Beheshti University of Medical Sciences, Tehran, Iran.; ^*b*^ Safety Promotion and Injury Prevention Research Center, Shahid Beheshti University of Medical Sciences, Tehran, Iran.; ^*c*^ Air Quality and Climate Change Research Center, Shahid Beheshti University of Medical Sciences, Tehran, Iran.; ^*d*^ Department of Public Health, School of Public Health and Safety, Shahid Beheshti University of Medical Sciences, Tehran, Iran.; ^*e*^ Department of Epidemiology, School of Public Health and Safety, Shahid Beheshti University of Medical Sciences, Tehran, Iran.; ^*f*^ Department of Neurobiology, Care Sciences and Society (NVS), H1, Division of Family Medicine and Primary Care, Huddinge, Stockholm, Sweden.

**Keywords:** Natural disasters, Spiritual rehabilitation, Spiritual health

## Abstract

**Background:**

Knowledge about the spiritual rehabilitation of affected people after disasters is scare. The objective of the present study is to identify the factors affecting the spiritual rehabilitation of affected people after natural disasters employing a systematic review study.

**Methods:**

The protocol of this review has been registered in the International Prospective Register of Systematic Review (PROSPERO) with the code CRD42021228552. Using MEDLIN (PubMed), Web of Science, Google Scholar, Embase, ProQuest, Scopus and ISC database as well as studies related to the research topic till the end of 2022. The Preferred Reporting Items for Systematic Reviews and Meta-Analyses (PRISMA) guidelines was used to find articles related to the research objective. Thematic content analysis then was used for concepts extraction.

**Results:**

This systematic review identifies factors affecting the spiritual rehabilitation of affected people after natural disasters.

**Conclusions:**

Both systematic review as well as qualitative study are essential in order to explore spiritual rehabilitation of affected people after natural disasters, while the current study was employed systematic review. It is expected that planners and policy-makers can use the extracted factors for improving the spiritual rehabilitation of people affected by natural disasters.

## Introduction

Spirituality has been part of human life since birth to death.^1^ Modern medicine in recent decades has shown a relationship between the body and the mind, and the incidence of diseases is not simply limited to the function of the physical body.^2^ Many studies have confirmed the significant relationship between spirituality and the level of human health.^3,4^ Spiritual health is the new and the fourth dimension of human health, which has been approved by the World Health Organization (WHO).^5^

Facing with the disasters, especially natural disasters, imposes pressure on the affected people and causes severe psychological and spiritual complications.^6,7^ When disasters occur, humans seek to understand the reality of disasters and try to manage them by the power of spirituality.^1^ Disruption in the spiritual beliefs of people affected by natural disasters and understanding a disaster as God's punishment, as well as the feeling of being rejected by God, reduces their level of spiritual health,^8^ which can cause mental disorders and increases suicide attempts among affected people.^9,10^ After Hurricane Katrina in 2005, which left 1,800 dead and 370,000 homeless, many affected people turned to religion and spirituality as a tool in order to cope with the psychological effects of the storm.^11^ Affected people by disasters, may seek religious acts such as prayer, and called it seeking spiritual support.^12^

The rehabilitation of the affected people is part of the recovery of disaster management cycle and it is attempted to reduce the long-term effects of diseases and physical, psychological, social and spiritual injuries as a result of the disasters.^13^ Those involved in the field of spiritual rehabilitation of affected people after natural disasters, including psychologists, psychiatrists, religious organizations, non-governmental organizations and clergy, should be aware of the factors affecting spiritual rehabilitation.^14^ in order to help organizations in charge of disaster management. However, tour best knowledge, no systematic study has been conducted to identify the factors affecting the spiritual rehabilitation of the affected people. Therefore, the objective of the present study was to develop a protocol to conduct a systematic review and identify factors affecting the spiritual rehabilitation of affected people after natural disasters.

## Methods 

***Protocol design***

This systematic review was conducted to find articles related to the objective of the research based on the Preferred Reporting Items for Systematic Reviews and Meta-Analyses (PRISMA) guidelines.^15^ The protocol of this review study is registered in the International Prospective Register of Systematic Review (PROSPERO) with the code CRD42021228552. Based on the PRISMA guidelines, the stages of search strategy, screening, and selection of studies, quality assessment and data extraction was performed, respectively. Accordingly, study selection, qualitative assessment and data extraction was performed by two researchers independently, and in case of disagreement, a decision was made through group discussion. Also, for the thematic content analysis, the six-step guide of Braun and Clarke were used. The six steps of thematic analysis covering familiarizing with the data, generating initial codes, searching for themes, reviewing themes, defining and naming themes, and producing the report was perforemed.^16^

***Search strategy and information sources***

In order to conduct a comprehensive search of MEDLIN (PubMed), Web of Science, Embase, ProQuest, Scopus, Google Scholar, and ISC databases, proceedings, key journals and reference list of selected articles and systematic review studies was used. In order to extract valid keywords, Medical Subject Heading (MeSH), keywords of related articles and consultation with scientific experts was used. Valid English keywords used in this study included "Spiritual Health", "Spiritual Rehabilitation", "Spiritual Care", "Spiritual well-being", "Spiritual Beliefs", "Spiritual Support", "Spiritual Therapy", "Spiritual Healing", "Spiritual Concepts", "Spiritual Indicators", "Spiritual Components", "Spiritual Counseling", "Spiritual Intervention", "Faith-based Health Care", "Spiritual", "Religion", "Faith-based", "Logo", "Rehabilitation", "Health", "Care", "Well-being", "Support", "Therapy", "Healing", "Counseling", "Intervention", "Disaster", "Crisis", "Emergency", "Catastrophe", "Accident", and "Event". First, the search writing structure was developed using keywords, operators and search fields based on PubMed, and then the search writing structure of other mentioned information sources was adapted based on PubMed. The search strategy is in English (Table 1).

**Table 1:** Search for electronic databases according to the Spiritual rehabilitation of affected people after natural disasters.

Database	Syntax
PubMed	((((((((((((((((("Spiritual Health"[Title/Abstract]) OR ("Spiritual Rehabilitation"[Title/Abstract])) OR ("Spiritual Care"[Title/Abstract])) OR ("Existential Care"[Title/Abstract])) OR ("Spiritual well-being"[Title/Abstract])) OR ("Spiritual Beliefs"[Title/Abstract])) OR ("Spiritual Sup-port"[Title/Abstract])) OR ("Spiritual Therapy"[Title/Abstract])) OR ("Spiritual Heal-ing"[Title/Abstract])) OR ("Spiritual Concept"[Title/Abstract])) OR ("Spiritual Indica-tor"[Title/Abstract])) OR ("Spiritual Component"[Title/Abstract])) OR ("Pastoral Care"[Title/Abstract])) OR ("Logo Therapy"[Title/Abstract])) OR ("Faith-Based Care"[Title/Abstract])) OR ("Spiritual Intervention"[Title/Abstract])) OR ("Spiritual Counsel-ing"[Title/Abstract])) AND (((((((((((((("Natural Disaster"[Title/Abstract]) OR (Earth-quake[Title/Abstract])) OR (Flood[Title/Abstract])) OR ("Man-made Disaster"[Title/Abstract])) OR ("Manmade Disaster"[Title/Abstract])) OR ("Technological Disaster"[Title/Abstract])) OR (Hurri-cane[Title/Abstract])) OR (Typhon[Title/Abstract])) OR (Tsunami[Title/Abstract])) OR (Storm[Title/Abstract])) OR (Landslide[Title/Abstract])) OR (Emergency[Title/Abstract])) OR (Cri-sis[Title/Abstract])) OR (Catastrophe[Title/Abstract])) AND (1000/1/1:2022/12/31[pdat])

***Eligibility criteria***

The inclusion criteria cover all studies focusing on the spiritual rehabilitation, including spiritual care, spiritual support, and spiritual counseling after natural disasters in English until the end of 2022. The study exclusion criteria are studies on the spiritual rehabilitation of technological (man-made) disasters, the spiritual rehabilitation of patients admitted to hospitals and other treatment and care centers, the spiritual rehabilitation of people with mental disorders, the spiritual rehabilitation of mothers with children with congenital abnormalities, the elderly living in Elderly care centers and other studies on the spiritual rehabilitation of affected people other than natural disasters.

***Study selection***

In order to manage the search results, all the articles was entered in EndNote X7. After excluding the repeated cases, the title and abstract of other articles screened based on the inclusion and exclusion criteria, and possible related articles identified. Next, two researchers independently read the full text of possible related articles; and finally the articles analyzed.

***Quality assessment***

At this stage, two researchers independently assessed the quality of the selected studies. The evaluation of the selected qualitative studies was done through the tool (Consolidated criteria for reporting qualitative research (COREQ). This tool included 32 questions, and the studies with a score above 25 included in the study.^17^ Then the Critical Appraisal Skills Programme (CASP) tool uses, including 10 questions with 3 options (Yes, No, Can't Tell), and articles with a score above 7 include.^18^ To evaluate studies that cannot be qualitatively evaluated, Modified STROBE uses. This tool contains 9 questions with 4 options (Yes, No, Not clear, Not applicable) and studies with a score above 6 include in the study.^19^

***Screening and data extraction***

Two researchers independently use a checklist covering corresponding author, year of study, place of study, study design, type of study and findings. Thematic content analysis used for data analysis. Based on the thematic content analysis, the text of the results also coded. For coding process, all the primary codes and concepts related to factors affecting the spiritual rehabilitation of affected people after natural disasters, which are extracted as main data from the studies. Then, two researchers investigate all the identified codes in terms of similarities and differences, and then similar codes were placed in a category and form a sub-theme. Finally, the draft summary of the findings discussed by authors and agreement was taken.

## Results

**Figure 1:**
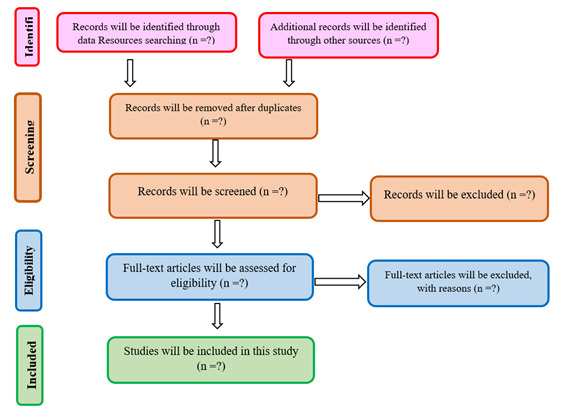
PRISMA flowchart of systematic literature review of the spiritual rehabilitation of affected people after natural disasters.

This systematic review identifies factors affecting the spiritual rehabilitation of affected people after natural disasters. (Figure 1). The study results, which is effective factors on the spiritual rehabilitation of affected people after natural disasters, reported as themes and sub-themes.

## Conclusion

Rehabilitating the spiritual dimension of the affected people after natural disasters is one of the undeniable necessities of the recovery of disaster management, which requires a ground through conducting relevant studies. Using this review, the effective factors in the spiritual rehabilitation of affected people by natural disasters extract. It is expected that effective steps can be taken to improve the level of spiritual health of the affected people by operationalizing the extracted factors. The effective factors extracted from this study can be used for the mitigation, to educate and improve the spiritual health level of society before disasters occur, and the preparedness, to formulate instructions and guidelines related to spiritual rehabilitation, as well as train those involved in spiritual rehabilitation.
